# Supplementary material for: Does Avian Coronavirus Co-Circulate with Avian Paramyxovirus and Avian Influenza Virus in Wild Ducks in Siberia?
Source: Viruses. 2023 May 7;15(5):1121. doi: 10.3390/v15051121 (PMC10222189; doi:10.3390/v15051121)
Supplement: Supplementary file 1 [file viruses-15-01121-s001.zip › viruses-2364505-supplementary.pdf]

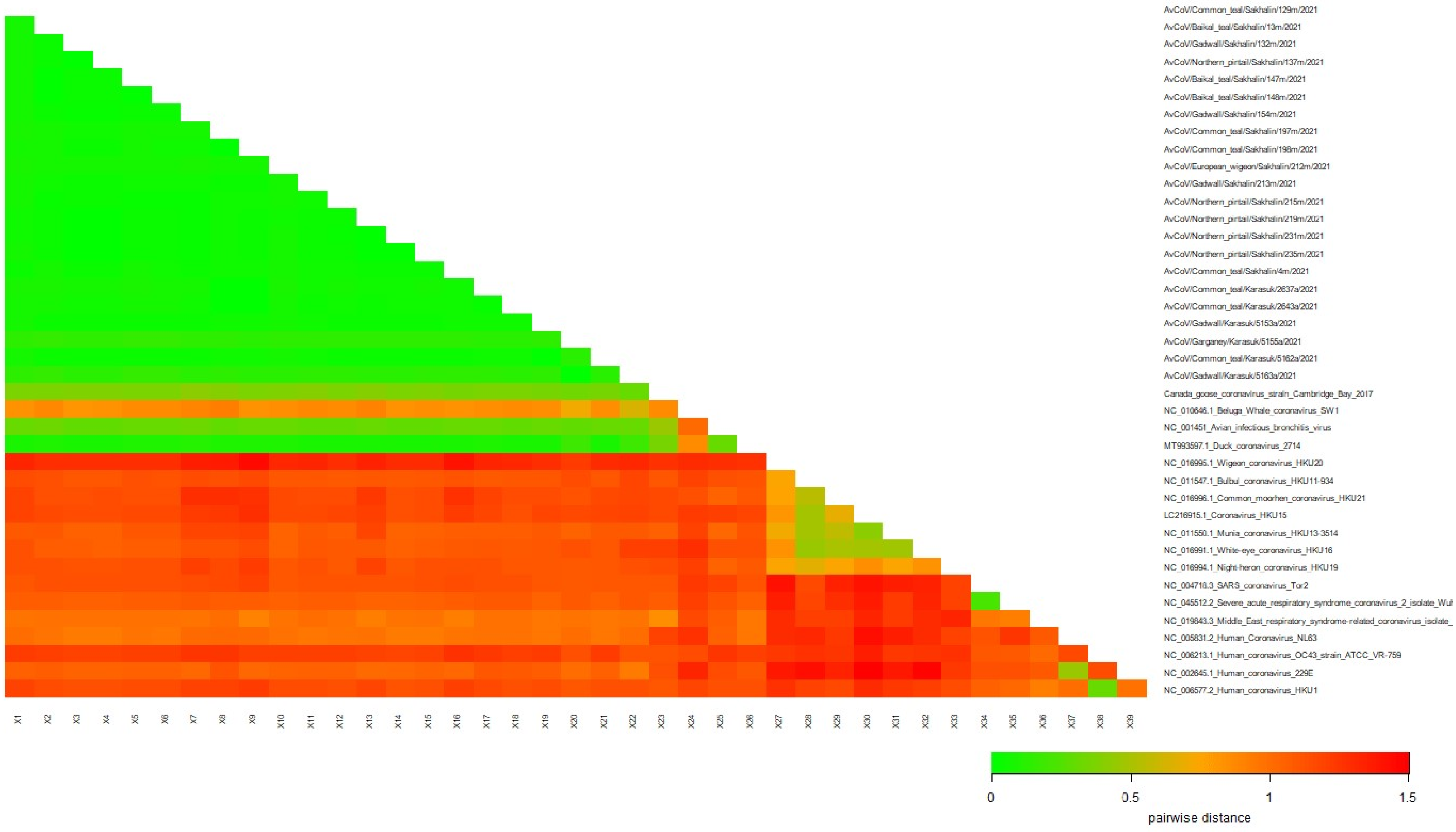

Figure S1. Heatmap with visualization of pairwise distances of nucleotide sequences of studied gammacoronaviruses and reference strains from four genera of coronaviruses: Alphacoronaviruses, Betacoronaviruses, Gammacoronavirus and Deltacoronavirus. The scale of pairwise distance is applied.
